# Supplementary material for: Complexome profiling of the Chlamydomonas psb28 mutant reveals TEF5 as an early PSII assembly factor
Source: Plant Cell. 2025 Mar 17;37(6):koaf055. doi: 10.1093/plcell/koaf055 (PMC12164590; doi:10.1093/plcell/koaf055)
Supplement: koaf055_Supplementary_Data [file koaf055_supplementary_data.zip › psb28_interactive.zip!/plots/intensity/qualityPlots/peptides/_Cre12.g483650.t1.2.html]

### Cre12.g483650.t1.2

Trivial name:   
SeqCoverage [median]: 22.70  
Mass: 50.45 kDa  
  
 

| Sequence | MissedCleavage | Mass [Da] | wtIntensity | muIntensity | Score |
| --- | --- | --- | --- | --- | --- |
| DIKPENLLVTVDGQVK | 1 | 1766.98 | 4072.14 | 19375.46 | 95.66 |
| DINVIK | 0 | 700.41 | 479.16 | 1492.08 | 104.11 |
| DKLGGGNFGITFEGLR | 1 | 1679.86 | 1453.45 | 609.80 | 108.63 |
| FESDATLGDALDGK | 0 | 1437.66 | 1779.53 | 13716.23 | 182.92 |
| LFNTELR | 0 | 891.48 | 2331.36 | 6596.55 | 86.87 |
| LQADDQGVTQR | 0 | 1229.60 | 6353.09 | 17266.67 | 192.44 |
| LSVSQALSHR | 0 | 1096.60 | 4525.58 | 18585.72 | 113.63 |
| QGVRQDFLK | 1 | 1089.59 | 0.00 | 391.75 | 66.27 |
| YSPPEELVMPQSFPR | 0 | 1775.86 | 0.00 | 2621.88 | 64.55 |
|  |  |  | 20994.3 | 80656.1 |  |
